# Experiences and attitudes of medical professionals on treatment of end-of-life patients in intensive care units in the Republic of Croatia: a cross-sectional study

**DOI:** 10.1186/s12910-022-00752-5

**Published:** 2022-02-16

**Authors:** Diana Špoljar, Marinko Vučić, Jasminka Peršec, Vlasta Merc, Tatjana Kereš, Radovan Radonić, Zdravka Poljaković, Višnja Nesek Adam, Nenad Karanović, Krešimir Čaljkušić, Željko Župan, Igor Grubješić, Jasminka Kopić, Srđan Vranković, Renata Krobot, Bojana Nevajdić, Mia Golubić, Štefan Grosek, Mirjana Kujundžić Tiljak, Andrija Štajduhar, Dinko Tonković, Ana Borovečki

**Affiliations:** 1 University Hospital Dubrava, Av. Gojka Šuška 6, 10000 Zagreb, Croatia; 2 Sestre Milosrdnice University Hospital Centre, Vinogradska cesta 29, 10000 Zagreb, Croatia; 3 University Hospital Centre Zagreb, Kišpatićeva ul. 12, 10000 Zagreb, Croatia; 4 Clinical Hospital Sveti Duh, Ul. Sveti Duh 64, 10000 Zagreb, Croatia; 5 University Hospital Centre Split, Spinčićeva 1, 21000 Split, Croatia; 6 Clinical Hospital Centre Rijeka, Krešimirova ul. 42, 51000 Rijeka, Croatia; 7 General Hospital ‘Dr. Josip Benčević’ Slavonski Brod, Ul. Andrije Štampara, 35000 Slavonski Brod, Croatia; 8 General Hospital Šibenik, Ul. Stjepana Radića 83, 22000 Šibenik, Croatia; 9 General Hospital Varaždin, Ul. Ivana Meštrovića 1, 42000 Varaždin, Croatia; 10 Marien Hospital Dusseldorf, Rochusstraße 2, 40479 Düsseldorf, Germany; 11 University Medical Centre Ljubljana, Bohoričeva 20, 1000 Ljubljana, Slovenia; 12 Andrija Štampar School of Public Health, School of Medicine, University of Zagreb, Johna Davidsona Rockfellera 4, 10000 Zagreb, Croatia

**Keywords:** Intensive care units, End-of-life care, End-of-life decision-making, Ethics

## Abstract

**Background:**

Decisions about limitations of life sustaining treatments (LST) are made for end-of-life patients in intensive care units (ICUs). The aim of this research was to explore the professional and ethical attitudes and experiences of medical professionals on treatment of end-of-life patients in ICUs in the Republic of Croatia.

**Methods:**

A cross-sectional study was conducted among physicians and nurses working in surgical, medical, neurological, and multidisciplinary ICUs in the total of 9 hospitals throughout Croatia using a questionnaire with closed and open type questions. Exploratory factor analysis was conducted to reduce data to a smaller set of summary variables. Mann–Whitney U test was used to analyse the differences between two groups and Kruskal–Wallis tests were used to analyse the differences between more than two groups.

**Results:**

Less than third of participants (29.2%) stated they were included in the decision-making process, and physicians are much more included than nurses (*p* < 0.001). Sixty two percent of participants stated that the decision-making process took place between physicians. Eighteen percent of participants stated that ‘do-not-attempt cardiopulmonary resuscitations’ orders were frequently made in their ICUs. A decision to withdraw inotropes and antibiotics was frequently made as stated by 22.4% and 19.9% of participants, respectively. Withholding/withdrawing of LST were ethically acceptable to 64.2% of participants. Thirty seven percent of participants thought there was a significant difference between withholding and withdrawing LST from an ethical standpoint. Seventy-nine percent of participants stated that a verbal or written decision made by a capable patient should be respected. Physicians were more inclined to respect patient’s wishes then nurses with high school education (*p* = 0.038). Nurses were more included in the decision-making process in neurological than in surgical, medical, or multidisciplinary ICUs (*p* < 0.001, *p* = 0.005, *p* = 0.023 respectively). Male participants in comparison to female (*p* = 0.002), and physicians in comparison to nurses with high school and college education (*p* < 0.001) displayed more liberal attitudes about LST limitation.

**Conclusions:**

DNACPR orders are not commonly made in Croatian ICUs, even though limitations of LST were found ethically acceptable by most of the participants. Attitudes of paternalistic and conservative nature were expected considering Croatia’s geographical location in Southern Europe.

**Supplementary Information:**

The online version contains supplementary material available at 10.1186/s12910-022-00752-5.

## Background

A certain percentage of patients in the intensive care units (ICUs) are at the ends of their lives and decisions about further diagnostic and treatment procedures are made accordingly. End-of-life decision-making is a process which involves physicians, nurses, patients and their families, and the goal is to decide whether to limit further (and which) treatments [1]. Both physicians and nurses find that most ethical dilemmas arise in their clinical practice relating to this subject [1, 2].

Studies have shown that withholding and withdrawing of treatment and shortening of the dying process were used less frequently in the southern European countries compared to the central or northern countries [3, 4]. It has also been shown that Catholic physicians and medical professionals are less inclined to follow a competent patient’s wish to refuse a treatment that might be lifesaving [5, 6].

Ethicus-2, a more recent prospective, multinational, observational study shows that the limitation of life-sustaining treatment (LST) occurs in about 12% of patients admitted to ICUs. This study confirms that treatment limitations are much more common in North America, Australia/New Zealand and Northern Europe than in Africa, Latin America and Southern Europe, and withholding LST is more common than withdrawing [7].

Many countries have specific guidelines which offer support and assistance to medical professionals in the decision-making process [8–14]. Many guidelines underpin the notion of a team of medical professionals making such decisions, and nurses as parts of that team, as they often have an intimate insight into patients' lives, are acquainted with their wishes and provide emotional support [15–18]. Physicians from northern European regions are of the opinion that nurses are more involved in the decision-making process than physicians from central and southern regions [19]. However, nurses feel they are not included in the decision-making process nor that their opinion is valued [18–22].

Croatian law bans euthanasia and physician-assisted suicide, while advance directives are not legally binding. Furthermore, according to laws on health care and patients’ rights, patients do not have the right to refuse treatment in case of mortal danger [23, 24]. There are no clearly defined national guidelines on end-of-life treatment and decision-making in Croatia. So far, an extensive, national survey on treatment of end-of-life patients has never been conducted in the Republic of Croatia, nor was Croatia ever included in a multinational survey of the type.

The aim of this research was to explore the professional and ethical attitudes and experiences of medical professionals on treatment of end-of-life patients in ICUs in the Republic of Croatia.

## Methods

A cross-sectional study was conducted using a questionnaire among physicians and nurses working in surgical, medical, neurological, and multidisciplinary ICUs in the total of 9 hospitals throughout Croatia, including 4 clinical centres, 2 clinical hospitals and 3 general hospitals. General hospitals in Croatia provide treatment for basic and simpler medical conditions and are less equipped than clinical hospitals, which are associated with a university and provide treatment for more complicated conditions. A clinical centre is the medical institution of the highest level.

The study was aimed at all nurses and medical doctors—specialists who work full time or perform overnight shifts in the ICU. Not all medical doctors working in the ICU are specialists in critical care. Residents and physicians who are temporarily working in selected ICUs were excluded.

The questionnaires were handed to the ICU directors who informed the staff about the aim and the conduction of the research. A quiet place was provided for all participants to fill out the questionnaires, which were then collected by the directors in a way which ensured participants’ anonymity and returned to the researcher. The ICU directors provided the total number of physicians and nurses working in the ICU to calculate the response rate.

The questionnaire was initially constructed by Groselj et al. for a cross-sectional, nation-wide study of experiences of Slovene ICU-physicians [25]. As Croatia and Slovenia are neighbouring countries that were once a part of the same federal republic and are now in a similar socio-economic situation, we opted for a questionnaire used there to make the comparisons easier.

The translations were conducted by registered translators and a back-translation was undertaken, meaning it was translated from Slovenian to Croatian, and back to Slovenian by another independent registered translator. The original Slovenian version and the back-translated Slovenian version were compared to check for quality and accuracy. It was comprehensively reviewed for linguistic, grammatical, and technical accuracy. Slight changes were made regarding the order of the questions, several questions were added, and the questionnaire was then validated for Croatian population.

The questionnaire consists of 4 parts with closed and open type questions (Additional File 1). The first part relates to general and demographic data, the second part explores the experiences of medical professionals regarding end-of-life decision-making and implementation of made decisions, while the third part explores the attitudes on the subject. The fourth part was intended for physicians only, as it consists of a made-up clinical scenario about a patient with a brain haemorrhage. The questionnaire was anonymous and took on average 15 min to complete.

A pilot study was conducted in a convenient sample of nurses and physicians in 2 different hospitals. Ethical clearance was obtained from the Ethics committee of the University of Zagreb—Medical school and from each participating hospital. The distribution and collection of the questionnaires took place from October 2018 to December 2019.

### Data analysis

The data from the questionnaires were compiled into an Excel sheet and all data were analysed using Python programming language. Descriptive statistics were conducted on all data. Information gathered in the open type questions were scarce and therefore excluded from further analysis. Cronbach’s alpha was used to measure internal consistency, and a coefficient of 0.70 or higher was considered acceptable. Exploratory factor analysis was conducted to reduce data to a smaller set of summary variables, and an oblique rotation (Promax) was used. Mann–Whitney U test was used to analyse the differences between two groups and Kruskal–Wallis tests were used to analyse the differences between more than two groups. Post-hoc analysis was conducted using the Holm–Bonferroni correction. Differences in categorical values were analysed with Yates’s chi-squared test. The significance level was set at *p* ≤ 0.05.

## Results

### Pilot study

The pilot study was conducted in a convenient sample of nurses and physicians in 2 different hospitals including 2 medical, 2 surgical and 2 neurological ICUs. The total response rate of the pilot study was 52.1%, the total number of participants was 208; 72.1% were female, 30.8% were physicians. Sixty-two and a half percent of physicians were anaesthesiologists, 23.4% were internal medicine physicians and 14.1% were neurologists. Since the questionnaire was not modified after the completion of the pilot study, the results from the pilot study were added to the results of the main study conducted in other hospitals.

### Characteristics of main study participants

The study was conducted in 18 ICUs in 9 different hospitals, including 3 medical, 5 surgical, 6 neurological and 4 multidisciplinary ICUs. The total response rate of all included participants was 51.5%, while physicians’ response rate was 63.1% and nurses’ 47.5%.

Total number of participants was 438; 75.8% were female, 31.3% were physicians. Seventy percent of physicians were anaesthesiologists, 13.1% were internal medicine physicians and 16.8% were neurologists. Participants’ mean age was 37.7 years (SD ± 11.5) with work experience on average 15.3 years (SD ± 108).

The other characteristics of study participants are listed in Table 1.

**Table 1:** Characteristics of study participants

	All N (%)	Physicians N (%)	Nurses N (%)	Male N (%)	Female N (%)
Vocation—education level					
Physician—specialist	137 (31.3)	–	–	60 (59.4)	77 (23.2)
Nurse—high school graduate	159 (36.3)	–	–	23 (22.8)	134 (40.4)
Nurse—college graduate	114 (26.0)	–	–	15 (14.9)	96 (28.9)
Nurse—university graduate	28 (6.4)	–	–	3 (3.0)	25 (7.5)
ICU type					
Surgical	219 (50.0)	66 (48.2)	153 (50.8)	56 (55.5)	161 (48.5)
Internal medicine	54 (12.3)	18 (13.1)	36 (12.0)	13 (12.9)	40 (12.1)
Neurological	75 (17.1)	23 (16.8)	52 (17.3)	13 (12.9)	62 (18.7)
Multidisciplinary	90 (20.6)	30 (21.9)	60 (19.9)	19 (18.8)	69 (20.8)
Work in ICU					
Every day	330 (75.3)	61 (44.6)	269 (89.4)	69 (68.3)	256 (77.1)
Occasional	84 (19.2)	75 (54.7)	9 (3.0)	31 (30.7)	53 (16.0)
Did not answer	24 (5.5)	1 (0.7)	23 (7.6)	1 (1.0)	23 (6.9)
Hospital type					
Clinical	384 (87.7)	117 (85.4)	267 (88.7)	91 (90.1)	289 (87.1)
General	54 (12.3)	20 (14.6)	34 (11.3)	10 (9.9)	43 (13.0)

### Experiences of medical professionals regarding end-of-life decision-making and implementation

Less than third of participants (29.2%) stated they were included in the decision-making process. Physicians are much more included than nurses (*p* < 0.001), and participants younger than 31 years and with total work experience less than 10 years are less included than their older and longer working colleagues (*p* < 0.001 in both cases). Sixty two percent of participants stated that the decision-making process took place between physicians, and only 23.4% of participants stated that nurses were involved in the decision-making. Two thirds of participants (66.7%) agreed that physicians were the ones who initiated the conversation about LST limitation, and only 2.5% said that nurses initiated such conversations.

Sixty percent of participants stated that verbal ‘do-not-attempt cardiopulmonary resuscitation’ (DNACPR) orders were given, and 59.1% state that verbal orders were given for other types of LST limitations in their ICUs. A DNACPR order was always respected by 67.4% of participants, with male participants respecting such orders more than female (*p* = 0.042).

When asked about the frequency of limitation of LST in their ICU, 18% of participants stated that DNACPR orders were frequently made in their ICUs, in contrast to 49.5% who stated that such decisions were rarely made; 13.7% of participants stated that therapy was frequently withheld, while 48.6% participants stated that such decisions were rarely made. A decision to withdraw inotropes and antibiotics was frequently made as stated by 22.4% and 19.9%, respectively. Withdrawal of mechanic ventilation was never performed as stated by 55.5%, the endotracheal tube was never removed as stated by 61.0%, and hydration was never stopped as stated by 69.0% of participants.

Half of the participants (49.1%) stated that family members/legal guardians were mostly or always included in the decision-making process. Detailed list of responses is shown in Table 2.

**Table 2:** Experiences regarding end-of-life decision-making and implementation

Questions/statements	Answers	All		Physicians		Nurses		Male		Female	
		N	%	N	%	N	%	N	%	N	%
Are DNACPR decisions made in your ICU?	Frequently	81	18.5	52	38.0	29	9.6	31	30.7	48	14.5
	Rarely	217	49.5	64	46.7	153	50.8	49	48.5	165	49.7
	Never	84	19.2	14	10.2	70	23.3	12	11.9	72	21.7
Are decisions to withhold LST made in your ICU?	Frequently	60	13.7	37	27.0	23	7.6	20	19.8	37	11.1
	Rarely	213	48.6	83	60.6	130	43.2	52	51.5	160	48.2
	Never	106	24.2	9	6.6	97	32.2	18	17.8	87	26.2
Are decisions to withdraw mechanical ventilation made in your ICU?	Frequently	14	3.2	5	3.7	9	3.0	6	5.9	7	2.1
	Rarely	120	27.4	36	26.3	84	27.9	25	24.8	92	27.7
	Never	243	55.5	87	63.5	156	51.8	60	59.4	183	55.1
Are decisions to withdraw endotracheal tube made in your ICU?	Frequently	22	5.0	2	1.5	20	6.6	3	3.0	19	5.7
	Rarely	90	20.6	32	23.4	58	19.3	21	20.8	68	20.5
	Never	267	61.0	95	69.3	172	57.1	67	66.3	197	59.3
Are decisions to withdraw inotropes made in your ICU?	Frequently	98	22.4	52	38.0	46	15.3	34	33.7	60	18.1
	Rarely	178	40.6	62	45.3	116	38.5	43	42.6	135	40.7
	Never	105	24.0	14	10.2	91	30.2	16	15.8	89	26.8
Are decisions to withdraw antibiotics made in your ICU?	Frequently	87	19.9	40	29.2	47	15.6	29	28.7	56	16.9
	Rarely	151	34.5	56	40.9	95	31.6	37	36.6	113	34.0
	Never	142	32.4	32	23.4	110	36.5	25	24.8	116	34.9
Are decisions to withdraw hydration made in your ICU?	Frequently	16	3.7	3	2.2	13	4.3	5	5.0	11	3.3
	Rarely	59	13.5	15	11.0	44	14.6	18	17.8	41	12.4
	Never	302	69.0	109	79.6	193	64.1	67	66.3	231	69.6
Are DNACPR decisions made and noted in the patient's medical records?	Yes. written	36	8.2	3	2.2	33	11.0	4	4.0	32	9.6
	Yes. verbal	266	60.7	92	67.2	174	57.8	64	63.4	198	59.6
	No	123	28.1	38	27.7	85	28.2	27	26.7	95	28.6
Are decisions to limit LST made and noted in the patient’s medical records?	Yes. written	56	12.8	11	8.0	45	15.0	12	11.9	44	13.3
	Yes. verbal	259	59.1	92	67.2	167	55.5	66	65.4	189	56.9
	No	104	23.7	34	24.8	70	23.3	21	20.8	82	24.7
Do you respect DNACPR decisions?	Always	295	67.4	93	67.9	202	67.1	78	77.2	213	64.2
	Rarely	85	19.4	26	19.0	59	19.6	14	13.9	70	21.1
	Never	33	7.5	8	5.8	25	8.3	4	4.0	29	8.7
Have you been included in LST limitation decision-making?	Yes	128	29.2	98	71.5	30	10.0	48	47.5	80	24.1
	No	287	65.5	31	22.6	256	85.1	51	50.5	231	69.6
LST limitation decision-making process includes ICU physicians and other physicians included in patient’s treatment	Very true	142	32.4	77	56.2	65	21.6	38	37.6	102	30.7
	True	131	29.9	46	33.6	85	28.2	38	37.6	92	27.7
	I cannot decide	41	9.4	5	3.7	36	12.0	6	5.9	35	10.5
	Not true	22	5.0	4	2.9	18	6.0	4	4.0	18	5.4
	Not true at all	61	13.9	3	2.2	58	19.3	10	9.9	50	15.1
LST limitation decision-making process includes ICU physicians and nurses	Very true	43	9.8	18	13.1	25	8.3	14	13.9	29	8.7
	True	60	13.7	21	15.3	39	13.0	18	17.8	42	12.7
	I cannot decide	70	16.0	16	11.7	54	17.9	14	13.9	55	16.6
	Not true	81	18.5	32	23.4	49	16.3	19	18.8	62	18.7
	Not true at all	119	27.2	35	25.6	84	27.9	28	27.7	88	26.5
Who initiates LST limitation discussion?	Physicians	292	66.7	106	77.4	186	61.8	69	68.3	220	66.3
	Nurses	11	2.5	2	1.5	9	3.0	4	4.0	7	2.1
	Family/legal guardians	21	4.8	5	3.7	16	5.3	2	2.0	19	5.7
Family members/legal guardians are included in LST limitation decision-making	Very true	90	20.6	17	12.4	73	24.3	16	15.8	74	22.3
	True	125	28.5	38	27.7	87	28.9	27	26.7	94	28.3
	Not true	127	29.0	51	37.2	76	25.3	37	36.6	90	27.1
	Not true at all	74	16.9	27	19.7	47	15.6	18	17.8	55	16.6

*ICU* Intensive care unit; *LST* Life-sustaining treatment; *DNACPR *Do-not-attempt cardiopulmonary resuscitation

### Attitudes of medical professionals regarding end-of-life decision-making and implementation

DNACPR orders and withholding/withdrawing of LST were ethically acceptable to 71.9% and 64.2% of participants, respectively. Thirty seven percent of participants stated they thought there was a significant difference between withholding and withdrawing LST from an ethical standpoint, with more participants working in general than in clinical hospitals (*p* = 0.020) having that opinion.

If the patient was incapacitated, 28.3% of participants stated that a team of physicians should decide about LST limitation, and 46.6% stated that such a decision should be made by a physician and the patient’s family/legal guardians.

Most of the participants (79.5%) stated that a verbal or written decision made by a capable patient should be respected. However, 55.2% of participants stated that they rarely or very rarely knew the patient’s wishes regarding LST limitation.

When asked about which aspects of the decision-making process should be respected, 80.8% of participants stated that good medical practice, 79% stated that patient’s interest, and 66% stated that patient’s autonomy should be respected.

Fifty eight percent of participants stated that family’s wishes, 50.2% stated that religious principles, and 68.3% stated that legal regulations should be respected. Seventy six percent of participants stated that advanced directives (AD) should also be respected, however 67.1% of participants have never encountered an AD in their practice, and only one participant (0.2%) stated they have encountered it often. Thirty eight percent and 13.5% of participants stated that treatment expenses and the need for ICU beds should be respected, respectively. Detailed list of responses is shown in Tables 3 and 4.

**Table 3:** Attitudes regarding end-of-life decision-making and implementation

Question	Answers	All		Physicians		Nurses		Male		Female	
		N	%	N	%	N	%	N	%	N	%
Do you think that withholding and withdrawing LST in end-of-life patients is ethically acceptable?	Yes	281	64.2	109	79.6	172	57.1	73	72.3	204	61.5
	No	21	4.8	3	2.2	18	6.0	1	1.0	20	6.0
	I cannot decide	124	28.3	22	16.1	102	33.9	25	24.8	98	29.5
Do you think there is a difference between withholding and withdrawing LST from an ethical standpoint?	Yes	163	37.2	53	38.7	110	36.5	38	37.6	124	37.4
	No	119	27.2	47	34.3	72	23.9	29	28.7	87	26.2
	I cannot decide	143	32.7	33	24.1	110	36.5	33	32.7	109	32.8
Do you think DNACPR decisions in end-of-life patients are ethically acceptable?	Yes	315	71.9	121	88.3	194	64.5	82	81.2	229	69.0
	No	31	7.1	3	2.2	28	9.3	4	4.0	27	8.1
	I cannot decide	86	19.6	13	9.5	73	24.3	15	14.9	70	21.1
Who should be included in LST limitation discussions if the patient is incapacitated?	Physician alone	4	0.9	0	0	4	1.3	1	1.0	3	0.9
	A group of physicians	124	28.3	54	39.4	70	23.3	36	35.6	86	25.9
	Physician and family members/legal guardians	204	46.6	36	26.3	168	55.8	39	38.6	165	49.7
	Hospital’s ethics committee	21	4.8	12	8.8	9	3.0	8	7.9	13	3.9
	The court	0	0	0	0	0	0	0	0	0	0
	Patient’s legal guardian based on patient’s AD	13	3.0	5	3.7	8	2.7	3	3.0	10	3.0
Written and/or verbal LST limitation decision made by a competent patient should be respected	Yes	348	79.5	107	78.1	241	80.1	75	74.3	268	80.7
	No	12	2.7	4	2.9	8	2.7	3	3.0	9	2.7
	I don’t know	69	15.8	23	16.8	46	15.3	22	21.8	47	14.2
How often are you acquainted with patients’ and their families' wishes about LST limitations?	Very frequently	22	5.0	5	3.7	17	5.7	4	4.0	17	5.1
	Frequently	111	25.3	50	36.5	61	20.3	33	32.7	77	23.2
	I cannot decide	48	11.0	11	8.0	37	12.3	10	9.9	38	11.5
	Rarely	184	42.0	54	39.4	130	43.2	39	38.6	143	43.1
	Very rarely	58	13.2	17	12.4	41	13.6	14	13.9	43	13.0
How often do you encounter AD in your practice?	Frequently	1	0.2	0	0	1	0.3	0	0	1	0.3
	Rarely	128	29.2	42	30.7	86	28.6	36	35.6	91	27.4
	Never	294	67.1	94	68.6	200	66.5	63	62.4	227	68.4

*LST* life-sustaining treatment; *DNACPR *do-not-attempt cardiopulmonary resuscitation; *AD* advance directives

**Table 4:** Attitudes regarding which aspects should be respected in LST limitation decision-making

The following aspects should be respected in LST limitation decision-making		All		Physician		Nurses		Male		Female	
		N	%	N	%	N	%	N	%	N	%
Good medical practice	I strongly agree	241	55.0	89	65.0	152	50.5	59	58.4	179	53.9
	I agree	113	25.8	31	22.6	82	27.2	29	28.7	84	25.3
	I cannot decide	39	8.9	9	6.6	30	10.0	9	8.9	29	8.7
	I disagree	9	2.1	1	0.7	8	2.7	1	1.0	8	2.4
	I strongly disagree	12	2.7	1	0.7	11	3.7	1	1.0	11	3.3
Patient’s interests	I strongly agree	225	51.4	96	70.1	129	42.9	55	54.5	167	50.3
	I agree	121	27.6	27	19.7	94	31.2	26	25.7	94	28.3
	I cannot decide	51	11.6	8	5.8	43	14.3	17	16.8	34	10.2
	I disagree	6	1.4	1	0.7	5	1.7	0	0	6	1.8
	I strongly disagree	15	3.4	4	2.9	11	3.7	3	3.0	12	3.6
Patient’s autonomy	I strongly agree	153	34.9	66	48.2	87	28.9	36	35.6	115	34.6
	I agree	136	31.1	41	29.9	95	31.6	30	29.7	104	31.3
	I cannot decide	84	19.2	17	12.4	67	22.3	22	21.8	62	18.7
	I disagree	18	4.1	5	3.7	13	4.3	7	6.9	11	3.3
	I strongly disagree	18	4.1	4	2.9	14	4.7	3	3.0	15	4.5
Treatment costs	I strongly agree	67	15.3	13	9.5	54	17.9	14	13.9	53	16.0
	I agree	100	22.8	26	19.0	74	24.6	21	20.8	77	23.2
	I cannot decide	91	20.8	35	25.6	56	18.6	29	28.7	62	18.7
	I disagree	87	19.9	29	21.2	58	19.3	20	19.8	67	20.2
	I strongly disagree	73	16.7	32	23.4	41	13.6	16	15.8	55	16.6
ADs	I strongly agree	209	47.7	67	48.9	142	47.2	41	40.6	165	49.7
	I agree	124	28.3	38	27.7	86	28.6	30	29.7	93	28.0
	I cannot decide	61	13.9	18	13.1	43	14.3	20	19.8	40	12.1
	I disagree	18	4.1	9	6.6	9	3.0	8	7.9	10	3.0
	I strongly disagree	12	2.7	4	2.9	8	2.7	2	2.0	10	3.0
Wishes expressed by the family/legal guardians	I strongly agree	94	21.5	16	11.7	78	25.9	10	9.9	83	25.0
	I agree	162	37.0	43	31.4	119	39.5	36	35.6	123	37.1
	I cannot decide	107	24.4	43	31.4	64	21.3	35	34.7	71	21.4
	I disagree	37	8.5	20	14.6	17	5.7	12	11.9	25	7.5
	I strongly disagree	22	5.0	13	9.5	9	3.0	8	7.9	14	4.2
Legal regulations	I strongly agree	159	36.3	64	46.7	95	31.6	34	33.7	124	37.4
	I agree	140	32.0	39	28.5	101	33.6	32	31.7	106	31.9
	I cannot decide	75	17.1	17	12.4	58	19.3	18	17.8	55	16.6
	I disagree	29	6.6	8	5.8	21	7.0	11	10.9	18	5.4
	I strongly disagree	15	3.4	6	4.4	9	3.0	4	4.0	11	3.3
Religious principles	I strongly agree	82	18.7	30	21.9	52	17.3	18	17.8	63	19.0
	I agree	138	31.5	44	32.1	94	31.2	27	26.7	110	33.1
	I cannot decide	130	29.7	32	23.4	98	32.6	37	36.6	91	27.4
	I disagree	36	8.2	17	12.4	19	6.3	10	9.9	26	7.8
	I strongly disagree	34	7.8	13	9.5	21	7.0	8	7.9	26	7.8
Need for beds in the ICU	I strongly agree	28	6.4	3	2.2	25	8.3	3	3.0	25	7.5
	I agree	31	7.1	10	7.3	21	7.0	10	9.9	21	6.3
	I cannot decide	65	14.8	10	7.3	55	18.3	14	13.9	50	15.1
	I disagree	89	20.3	32	23.4	57	18.9	28	27.7	61	18.4
	I strongly disagree	205	46.8	80	58.4	125	41.5	46	45.5	156	47.0

*ICU* intensive care unit; *LST* life-sustaining treatment; *AD* advance directives

### Exploratory factor analysis

In order to reduce data to a smaller set of summary variables Exploratory factor analysis was conducted. We divided the data into two subsets: the first included the Likert type questions where the maximum value was 5 (1 = strongly disagree–5 = strongly agree), and the second subset included questions where the maximum value was 3 or 4. Barlett’s test of sphericity was significant (*p* < 0.001) for both subsets of data. The Kaiser–Meyer–Olkin measure of sample adequacy was 0.7330 for the first and 0.6962 for the second subset of data, indicating that the sampling is adequate for factor analysis, however middling.

The sum of squared loadings, proportional and cumulative variance, shown in Table 5, provide more information on relevancy and the information provided by the factors. Due to the middling results of KMO, the factors have moderate contribution to the explained variance.

**Table 5:** Sum of squared loadings, proportional variance, and cumulative variance for each factor

Factor	Sum of squared loadings	Proportional variance (%)	Cumulative variance (%)
Respecting patients’ wishes	2.5033	13.9	13.9
Respecting religious and cultural principles	1.5305	8.5	22.4
Paramedical aspects of decision-making	1.4549	8.1	30.5
Decision-making process including nurses	0.9838	5.5	36
Common withdrawal of therapies	2.1067	12.4	12.4
Uncommon withdrawal of therapies	1.5147	8.9	21.3
Disagreement in decision-making	1.2628	7.4	28.7
Liberal attitudes towards LST limitation	1.0616	6.2	35

The exploratory factor analysis yielded 8 different factors. One factor has subsequently been reduced to one question. All of the questions in that factor were related to the topic of parties included in the decision-making process. However, due to the way the questions were formulated, it was not possible to analyse them as one factor. Therefore, we decided to focus on the question pertaining to the inclusion of nurses in the decision-making process.

Factor were analysed according to the hospital type, ICU type, age, sex, vocation, level of education, total work experience, ICU work experience and specialisation.

List of factors, cumulative variance explained by each factor, comprising questions and the sum of squared loading are shown in Table 6.

**Table 6:** List of factors, cumulative variance, comprising questions and the sum of squared loadings

Factor name (cumulative variance explained by each factor)	Comprising questions	Sum of squared loadings
Respecting patients’ wishes (13.9%)	Patients’ interests should be respected in LST limitation decision-making	0.8173
	Patients’ autonomy should be respected in LST limitation decision-making	0.6914
	AD should be respected in LST limitation decision-making	0.6039
	Good medical practice should be respected in LST limitation decision-making	0.5534
	Legal regulations should be respected in LST limitation decision-making	0.5242
	Families’ wishes should be respected in LST limitation decision-making	0.4182
	How often are you acquainted with patients’ and families’ wishes?	0.1061
Respecting religious and cultural principles (22.4%)	Religious and cultural principles expressed by the patient or family should be respected	1.0298
	Religious principles should be respected in LST limitation decision-making	0.4488
	Do you think AD are helpful in the decision-making process?	0.3148
	Religious and cultural principles expressed by the physician should be respected	0.2408
Paramedical aspects of decision-making (30.5%)	Need for beds in the ICU should be respected in LST limitation decision-making	0.7197
	Treatment costs should be respected in LST limitation decision-making	0.6231
	Is health care resource allocation important in decision-making?	0.5253
Decision-making process including nurses (36%)	LST limitation decision-making process includes ICU physicians and nurses	0.6929
Common withdrawal of therapies (12.4%)	Are decisions to withdraw antibiotics made in your ICU?	0.7639
	Are decisions to withdraw inotropes made in your ICU?	0.7477
	Are decisions to withhold LST made in your ICU?	0.7069
	Are DNACPR decisions made in your ICU?	0.6075
Uncommon withdrawal of therapies (21.3%)	Are decisions to withdraw endotracheal tube made in your ICU?	0.876
	Are decisions to withdraw mechanical ventilation made in your ICU?	0.6829
	Are decisions to withdraw hydration made in your ICU?	0.4667
	Do you agree that hydration should be withdrawn in end-of-life patients?	0.0654
Disagreement in decision-making (28.7%)	How often is agreement between physicians not achieved?	0.7366
	How often is agreement between physicians and family/legal guardians not achieved?	0.6122
	Have you ever disagreed with the method of LST limitation?	0.5269
	Have you ever refused to be a part of decision-making discussion or implementation?	0.1134
	Do you think there is a difference between withholding and withdrawing LST from an ethical standpoint?	0.0628
Liberal attitudes towards LST limitation (35%)	Do you think that withholding and withdrawing LST in end-of-life patients is ethically acceptable?	0.694
	Do you think DNACPR decisions in end-of-life patients are ethically acceptable?	0.5893
	Do you respect DNACPR decisions?	0.2799
	Do you think LST limitation is the same from an ethical standpoint in the adult patients who are brain dead, terminally ill or in a vegetative state?	0.1232

*ICU* intensive care unit; *LST* life-sustaining treatment; *DNACPR* do-not-attempt cardiopulmonary resuscitation

Analysis of the factors showed that physicians were more inclined to respect patient’s wishes then nurses with high school education (*p* = 0.038), however nurses with high school (*p* < 0.001), college (*p* = 0.005) and university education (*p* = 0.003) were more inclined to respect religious and cultural principles than physicians.

Participants younger then 31 years are more inclined to respect religious and cultural principles than those aged 32–44 (*p* = 0.022).

A higher inclination towards paramedical aspects of decision-making process was noted in neurological and multidisciplinary ICUs compared to surgical (*p* < 0.001 and *p* = 0.044, respectively), neurologists compared with anaesthesiologists (*p* = 0.019), medical professionals aged 45–57 years in comparison to those aged less than 31 years (*p* = 0.003), male participants compared to female participants (*p* = 0.001), and physicians compared to nurses with high school (*p* < 0.001), college (*p* < 0.001) and university education (*p* = 0.014).

Analysis showed that nurses were more included in the decision-making process in neurological more than in surgical, medical, or multidisciplinary ICUs (*p* < 0.001, *p* = 0.005, *p* = 0.023 respectively). They were also more included in surgical than in medical ICUs (*p* = 0.005).

Male participants and physicians were more prone to withholding of LST, instigating DNACPR orders and withdrawing of antibiotics and inotropes than female participants and nurses with college and university education (*p* < 0.001 in all cases).

Withdrawal of mechanical ventilation, endotracheal tubes and hydration was more common in clinical compared to general hospitals (*p* = 0.016), and in neurological ICUs compared to surgical (*p* = 0.031), medical (*p* = 0.005), or multidisciplinary (*p* = 0.003).

Male participants in comparison to female (*p* = 0.002), physicians in comparison to nurses with high school and college education (*p* < 0.001 in both cases), and medical professionals aged 32–57 years in comparison to those aged less than 31 years (*p* < 0.001) displayed more liberal attitudes about LST limitation.

No significant differences were noted among the groups regarding disagreement in the decision-making process.

## Discussion

This is the first study to assess the experiences and attitudes of medical professionals working in ICUs in Croatia on the treatment of end-of-life patients. Our results show that LST limitations occur less frequently than in other countries, even though they were found ethically acceptable by most of the participants. This may be caused by the discrepancy between the attitudes created by the reality ICU medical professionals witness on a daily basis and what is allowed by the law. Croatia is a mainly catholic country [26] and paternalistic and conservative attitudes are expected considering geographical location in Southern Europe, as found by previous studies [3–7].

American Society of Critical Care Medicine has stated back in 1989 that LST limitations are ethically appropriate in certain cases [27]. More recent research conducted in the Netherlands, Switzerland, Denmark, Sweden, Belgium and Italy showed that 23–51% of patients died after a decision to limit LST has been made [28], while Ethicus-2 study showed that such a decision is made in as much as 12% of patients admitted to ICU and in almost 81% of the study population, which included patients who died in the ICU. It also showed that withholding of LST occurred in 44% and withdrawing of LST occurred in 36% of the study population [7]. A study conducted in the ICUs in the city of Milan, Italy, showed that 73% of physicians indicated that DNACPR orders were used in their ICU [29].

Our research shows that LST limitation does not occur often, as only 18% of participants stated that DNACPR orders were frequently made in their ICUs, and only 13% of participants stated that therapy was frequently withheld. Study of experiences in Slovene ICUs showed a DNACPR orders are made more commonly than decisions to withhold treatments [25]. However, 67% of Slovene physicians frequently make DNACPR decisions, as opposed to 38% of Croatian physicians.

Studies conducted in Germany, Italy and Denmark also showed that DNACPR orders are made often and are more frequent than limitation of antibiotics and vasoactive medications [29–31]. The results of a multicentric study conducted in Spain are consistent with previous studies which showed that, in comparison to Northern European countries, DNACPR decisions were less frequently noted in the patient’s medical documents and less LST limitation decision were made [32].

Even though withdrawal of mechanical ventilation, endotracheal tubes and hydration is not very common in Croatian ICUs, it is more common in clinical compared to general hospitals. Research conducted by Bach showed that university-based intensivists were more prone to instigating DNACPR orders and withdrawing LST than community-based intensivists [33].

Most participants in our study found that DNACPR orders and withholding/withdrawing of LST were ethically acceptable, and DNACPR orders were always respected by 67.4% of participants. Thirty seven percent of participants stated that there was a difference between withholding and withdrawing LST from an ethical standpoint. Many end-of-life guidelines purport that there is no ethical difference between withholding and withdrawing of LST, which is supported by ethical principles of professional duty, beneficence, nonmaleficence and autonomy [15].

Nevertheless, almost half of participants in a study conducted in Milanese ICUs stated that there is a difference [29]. Studies exploring nurses’ attitudes also found that about half of nurses find that withholding of LST is not morally the same as withdrawal [22, 34, 35]. Seventy percent of participants in a study conducted in tertiary care hospitals in Sri Lanka responded they found withholding LST more comfortable then withdrawing it [36].

Involvement of nurses in end-of-life decision-making process is a widely accepted attitude. Nonetheless, multiple studies confirm that nurses are not sufficiently included. Our results show that only 28% of physicians and 21% of nurses stated that nurses were included in the decision-making, while almost 50% of physicians stated they did not include nurses. Around 60% of Slovene intensivists stated they never included nurses in such decisions, and only 5% stated they were always included [25]. Half of participants in a study conducted in Germany [30] and 90% of participants in Portugal [37] stated that nurses were not included in the decision-making. Similar results were found in studies conducted in Italy and Hong Kong [29, 38]. Studies exploring nurses’ attitudes and experiences on the matter found that nurses thought they were not included, and their opinions were not esteemed [18–21, 39].

A study conducted in France in 2003. showed that, despite the opinion that nurses should be included in the decision-making process, 50% of physicians and only 27% of nurses stated it occurred in practice [40]. Another study conducted in France after a law allowing withholding and withdrawing of LST was passed, showed an improvement [41]. This is an encouraging example of how a change of legal aspects can positively affect everyday practice.

Apart from not being sufficiently included in the decision-making process, nurses are not adequately active in initiating discussions about LST limitation. Our research showed that only 2% of physicians and 3% of nurses stated it were nurses who initiated such discussions. This is confirmed by other studies with similar findings [19, 34, 42]. Badir suggests the fact that nurses fail to initiate LST limitation discussions is a source of ethical concern, as in ensuring quality end-of-life care it is important that nurses learn and meet the needs and expectations of patients who seek a dignified death [22].

Analysis of the factors in our study showed that physicians were more inclined to respect patient’s wishes then nurses with high school education. Other research showed that more experienced physicians were more inclined to take patient’s wishes in account in end-of-life decision-making [29], and that more male than female physicians found patient’s wishes to be the most important criterion in LST limitation decision-making [37]. Our research did not find such differences.

Nevertheless, Croatian ICU nurses of all levels of education were more inclined to respect religious and cultural principles than physicians. A study from South Africa points to the same direction, as 75% and 63% of nurses declared that patient’s and families’ religious beliefs, respectively, are important in the decision-making process [34].

Our study shows that most of the participants found patient’s interests and autonomy to be an important aspect to be considered when making end-of-life decisions. Most of them also stated that a verbal or written decision made by a capable patient should be respected. However, 55.2% of participants stated that they rarely knew the patient’s wishes regarding LST limitation. Therefore, a conclusion can be extracted that Croatian medical professionals find autonomy to be an important principle, but they are not adequately informed about patient’s wishes, which casts a doubt on whether those wishes are actually respected. Ethical principles of autonomy, privacy and nonmaleficence underpin the significance and importance of respecting patient’s wishes. End-of-life guidelines affirm the pertinence of encouraging patients to express their will and wishes while capable for it to be respected once they become incompetent [15]. Medical professionals should motivate patients to express their opinions and wishes [43].

Seventy six percent of participants in our research stated that AD should be respected, but it is almost never encountered in their practice. A study conducted in Slovene ICUs also found that physicians rarely encountered AD [25], and a study from Milan showed that 70% of physicians were not acquainted with the notion of AD [29].

This study has several limitations. The total response rate was not as high as expected and there is a possibility of bias, as it may be that most of the participants have a special interest in the topic and were more inclined to fill out the questionnaire. The research was not conducted in all the hospitals in the Republic of Croatia even though it did cover all geographic regions, and residents were not included. All steps were taken to protect participant anonymity, however, since certain actions described in the questionnaire are not allowed according to Croatian law, it is possible that some participants adjusted their responses.

## Conclusion

Our study has found that DNACPR orders are not commonly made in Croatian ICUs, even though limitations of LST were found ethically acceptable by most of the participants. It has also shown the inadequate involvement of nurses in the decision-making process. The results have confirmed our expectations of paternalistic and conservative attitudes considering Croatia’s geographical location in Southern Europe.

This was the first study about medical professionals’ attitudes and experiences on treatment of end-of-life patients in ICUs in Croatia and has provided an insight into the current state of the issue. In addition, it confirms the findings of previous studies, and it can be used to help evaluate and compare the situation in other neighbouring countries which are in a similar socio-economic situation.

This type of research should be repeated in the future to assess possible changes, and to provide more data which would help in making and shaping the guidelines and legally binding policies on treatment of end-of-life patients in Croatia.

## Supplementary Information

**Additional file 1.** The Questionnaire. The questionnaire used in this research, translated in English.

## Data Availability

All data generated or analysed during this study are included in this published article [and its Additional file 1].

## Abbreviations

ICU: Intensive care unit
LST: Life-sustaining treatment
DNACPR: Do-not-attempt cardiopulmonary resuscitation
AD: Advance directives
