# Supplementary material for: Experiences and attitudes of medical professionals on treatment of end-of-life patients in intensive care units in the Republic of Croatia: a cross-sectional study
Source: BMC Med Ethics. 2022 Feb 16;23:12. doi: 10.1186/s12910-022-00752-5 (PMC8851755; doi:10.1186/s12910-022-00752-5)
Supplement: Supplementary file 1 — Additional file 1. The Questionnaire. The questionnaire used in this research, translated in English. [file 12910_2022_752_MOESM1_ESM.doc]

**ETHICAL ATTITUDES in decision-making when LIMITING LIFE-PROLONGING tREATMENT IN TERMINALLY ILL PATIENTS IN INTENSIVE CARE UNITS**

THIS SURVEY IS ANONYMOUS

I. DATA

- Hospital _________________________________________________________
- Type of ICU:
  1. Anaesthesiology
  2. Internal/Medical
  3. Paediatric
  4. Neurology
  5. Multi-discipline
- Age (in years) __________________
- Sex (please circle) M / F
- Status:

1. Specialist doctor
2. Nurse
3. Senior nurse
4. Graduate senior nurse

- How many years have you worked as a physician/nurse? ______________________
- How many years have you worked in the ICU? ______________________________
- What is your specialization (for doctors)?:__________________________________
- Work in the ICU:

1. Regular - I work in the ICU every day
2. I occasionally work in the ICU - only when on duty or when there is a shortage of doctors in the ICU

- Do you know who chairs the Ethics Committee at your hospital?

1. Yes
2. No

- Do you know who to refer to if an ethical dilemma arises?

a) Yes

b) No

- Have you ever referred to the Ethics Committee of your institution?

a) Yes

b) No

II. EXPERIENCE - IMPLEMENTATION

In the following questions, we are interested in your involvement and participation in restricting treatment in terminally ill patients.

**1.) What form of limitation of treatment do you make decisions about in your ICU? Do the following statements apply? (circle)**

1. No resuscitation using cardiopulmonary resuscitation methods:

Several times / rarely / never

1. Refraining from initiating treatment – *withholding:*

Several times / rarely / never

1. Discontinuing treatment - *withdrawing*:

- Artificial respiration

Several times / rarely / never

- Removing endotracheal intubation

Several times / rarely / never

- Inotropes

Several times / rarely / never

- Antibiotics

Several times / rarely / never

- Hydration

Several times / rarely / never

- Other (describe): _____________________________________

**2.) Are decisions made and recorded in your ICU about not using cardiopulmonary resuscitation methods in terminally ill patients?**

a) Yes, in writing

b) Yes, orally

c) No

**3.) Do you respect instructions on not using cardiopulmonary resuscitation methods?**

1. Always
2. Rarely
3. Never

**4.) Do you use oral or written instructions on limiting treatment to prolong life?**

1. Yes, oral
2. Yes, written*
3. Instructions for limiting treatment are not used

*If YES: Do you have a separate form? (You can enclose it with the completed questionnaire)

- Yes
- No

**5.) Have you been involved in deciding on limiting procedures to prolong life in terminally ill patients?**

a) Yes

• How many times this year (2018)?_________________

- since 2013 – 2018______________

• What was the most frequent reason for the decision?:

- Brain death

-Terminal illness - Vegetative state

- Other __________________________________

b)No

**6.) How did the discussion on limiting procedures to prolong life begin?**

a) At the initiative of a doctor

b) At the initiative of a nurse

c) At the initiative of the family/legal guardian

d) Other __________________________________

**7.) How does decision-making work in your ICU? I decide (circle):**

(1= Extremely true; 2 = True; 3 = I don’t know; 4 = Not true; 5 = Not true at all)

| I decide alone | 1 | 2 | 3 | 4 | 5 |
| --- | --- | --- | --- | --- | --- |
| In a council with doctors from the ICU and consultants | 1 | 2 | 3 | 4 | 5 |
| In a council with doctors and nurses from the ICU | 1 | 2 | 3 | 4 | 5 |
| I ask the hospital's Ethics Committee for an opinion | 1 | 2 | 3 | 4 | 5 |

**8.) Is the following statement true: "Family members or the legal guardians are involved in decision-making"?**

1. Always true
2. Mainly true
3. Rarely true
4. Never true

**9.) What is the further procedure for your decision-making if no agreement is reached amongst the doctors? (describe)**

**________________________________________________________________________________________**

**10.) What is the further procedure for your decision-making if no agreement is reached between the doctors and the family or legal guardian? (describe)**

**________________________________________________________________________________________**

**11.) How often is no agreement reached between doctors?**

1. Often
2. Rarely
3. Never

**12.) How often is no agreement reached between the doctors and the family or the legal guardian?**

1. Often
2. Rarely
3. Never

**13.) Has it ever happened that you sometimes do not agree with how life-prolonging treatment is limited?**

1. Often
2. Rarely
3. Never

**14.) Have you ever refused to participate in decision-making to limit life-prolonging treatment, or to implement it?**

1. Yes

- Often
- Rarely
- Never

1. No

*If YES, describe why:

______________________________________________________________________

**15. How much time passes from making a decision until treatment is withdrawn?**

1. Immediately
2. Immediately as soon as the decision is made and the family has agreed to it
3. About 6 hours
4. About 24 hours
5. More than 24 hours

- If more than 24 hours: What is the reason for this delay? (describe):

_____________________________________________________________________________

III. OPINIONS

In the following questions we are interested in your opinions on limiting treatment to prolong the life of terminally ill patients There are no right or wrong answers

**1.) What do you understand by the term "terminally ill patient"?**

a) A patient for whom it is presumed that they will die within the next 12 months, which includes patients whose death is unavoidable in the next several hours or days, due to deterioration of their basic illness or sudden deterioration of their health

a) A patient for whom it is presumed that they will die within the next several hours or days, due to the deterioration of their basic illness or sudden deterioration of their health

c) Other (describe): __________________________________________________________________

**2.) What in your opinion is the definition of the expression "*withholding treatment”*?**

1. No new medication or other treatment measures are added, only continuing what the patient is already receiving
2. Medication is discontinued that does not improve the patient's condition
3. The patient is not resuscitated using cardiopulmonary measures, but the use of all other treatment continues
4. All medication except liquids is discontinued
5. Other (describe) _____________________________________________________

**3.) What in your opinion is the definition of the expression "*withdrawing treatment”*?**

1. All medication except analgesics and sedatives is discontinued
2. All medication is discontinued and the patient is disconnected from the respirator if they are on a respirator
3. All medication is discontinued, the patient is disconnected from the respirator if they are on a respirator, and endotracheal intubation is removed
4. All medication is discontinued and supportive care is halted (dialysis, extracorporeal circulation etc.)
5. other (describe) ______________________________________________________________

**4.) In your opinion, is the limitation of life-prolonging procedures in the sense of withholding treatment and withdrawing treatment in terminally ill patients an ethically acceptable decision?**

1. Yes
2. No
3. I cannot decide

**5.) Is there in your opinion from the ethical point of view a difference between withholding and withdrawing treatment?**

1. Yes
2. No
3. I cannot decide

**6.) In your opinion, is a decision not to resuscitate using cardiopulmonary resuscitation methods in terminally ill patients an ethically acceptable decision?**

1. Yes
2. No
3. I cannot decide

**7.) Do you agree that when limiting life-prolonging procedures in terminally ill patients hydration should also be discontinued?**

1. Yes
2. No
3. I cannot decide

*If YES: Do you agree even in cases when it is expected that this could be the direct cause of the death of the terminally ill patient?

1. Yes
2. No
3. I cannot decide

**8.) In your opinion, is decision-making on limiting life-prolonging procedures the same from an ethical point of view in the following cases: in the case of an adult patient in whom brain death has been established or who is terminally ill, or who is in a persistent vegetative state?**

1. Yes
2. No*
3. I cannot decide

*If NO, in which case would the decision differ from the others mentioned? (describe):_____________________________________________________________

**9.) How far do you agree with the following statement: "When deciding on limiting life-prolonging procedures, it is necessary to respect the following aspects..." Please circle the answer that applies to you.**

1= I strongly agree, 2= I agree, 3= I cannot decide, 4= I disagree, 5= I strongly disagree

| Good medical practice | 1 | 2 | 3 | 4 | 5 |
| --- | --- | --- | --- | --- | --- |
| The patient's interests | 1 | 2 | 3 | 4 | 5 |
| The patient's autonomy | 1 | 2 | 3 | 4 | 5 |
| The costs of treatment | 1 | 2 | 3 | 4 | 5 |
| A written document (living will) expressing the patient's wishes | 1 | 2 | 3 | 4 | 5 |
| The wishes of the family/legal guardian | 1 | 2 | 3 | 4 | 5 |
| Legislation | 1 | 2 | 3 | 4 | 5 |
| Religious principles | 1 | 2 | 3 | 4 | 5 |
| The need for an empty bed in the ICU | 1 | 2 | 3 | 4 | 5 |

**10.) In your opinion, is decision-making on limiting life-prolonging procedures the same from an ethical point of view if the patient from the previous question is a child?**

1. Yes
2. No*
3. I cannot decide

*If NO, in which case would the decision differ from others mentioned? (describe):_____________________________________________________________

**11.) Is consideration of the distribution of the insufficient resources of the health service in your opinion of importance when deciding on the further treatment of a terminally ill patient? (circle)**

Very important / Important / I cannot decide / Not important /Not important at all

**12.) Who in your opinion should decide on the limitation of life-prolonging procedures for a terminally ill patient if the patient is incapable of deciding for themselves?**

a) the doctor alone

b) a doctors' council

c) the doctor + the family/ legal guardian

d) the hospital's ethics committee

e) a court

f) the patient's legal guardian on the basis of an advance decision (living will) by the patient to withdraw intensive treatment

**13.) Do you believe that it is necessary to respect the written or verbally expressed decision of the patient to limit life-prolonging procedures if the patient is capable of making a decision?**

a) Yes

b) No

c) I don't know

**14.) Is the following statement true: "The different religious/cultural beliefs expressed by the patient or legal guardian should be respected"?**

Extremely true/ true / I cannot decide / Not true / Not true at all

**15.) Is the following statement true: "The different religious/cultural beliefs expressed by the doctor should be respected"?**

Extremely true/ true / I cannot decide / Not true / Not true at all

**16.) How often have you learned about the wishes of the patient or their family (legal guardian) about the continuation of active treatment or limitation of life-prolonging procedures in terminally ill patients?**

Very often / Often / I cannot decide / Rarely / Very rarely

**17.) Do you feel that an Advance Decision (Living Will) expressing the patient's wishes would help in decision-making in relation to treating terminally ill patients?**

Extremely true/ true / I cannot decide / Not true / Not true at all

**18.) How often do you come across an Advance Decision (Living Will) expressing the patient's wishes in practice?**

a) Often

b) Rarely

c) Never

**19.) Do you believe that you personally have legal/ criminal responsibility in making and executing decisions on limiting life-prolonging procedures?**

1. Yes
2. No
3. Don't know

IV. CLINICAL SCENARIO - A TERMINALLY ILL PATIENT - only for doctors

In the following imaginary example, we are interested in your decisions relating to terminally ill patients - please circle one answer.

A forty-year old patient has been admitted to the ICU after a brain haemorrhage with intracerebral and interventricular bleeding, oedema and hematocephalus. After admission he has generalized seizures, that do not stop despite administration of anti-epileptics. When the seizures finally stop, the patient has dilated, non-reactive pupils.

The doctor on duty calls the consultant neurologist or neurosurgeon. On the basis of an examination, brain CT and the absence of reflexes, and the non-reactive pupils, it is believed that there has been irreversible brain damage, with the greatest probability that the patient will be in a persistent vegetative state. The patient's blood pressure and pulse drop, and additional treatment is needed: the addition of new catecholamines and a higher dose of catecholamines to maintain blood pressure, and the immediate insertion of ventricular drainage.

The legal representative - his wife - wants everything to be done to keep him alive. The patient has not written any form of advance decision (living will) stating that treatment should be continued in the case of a severe condition that would end in severe disability.

**1.) How would you make a decision about continuing treatment in the ICU?**

- 1. I would decide alone
  2. I would decide after consultation with a neurosurgeon and neurologist who are acquainted with the patient's condition
  3. I only would decide after consulting with a fellow intensive care doctor
  4. I would wait for the next day and after the doctor's report make a decision together with my colleagues in relation to continuation of treatment
  5. I would ask the hospital's Ethics Committee for an opinion
  6. Other (describe): _________________________________________________

**2.) You have decided that the surgeon should urgently insert external ventricular drainage. After surgery, the patient's cardiovascular condition stabilizes, but he does not react, he has no reflexes, his pupils are dilated and non-reactive. How will you proceed when the patient returns to the ICU?**

1. You issue a *Do Not Resuscitate* (DNR) order.
2. You withhold introduction of new treatment
3. You withdraw treatment
4. You continue full treatment

**3.) You have decided that after insertion of the external ventricular drainage you will withdraw treatment. What does this mean in your institution?**

1. You discontinue medication to maintain blood pressure
2. You discontinue artificial respiration and remove the endotracheal intubation
3. You discontinue all medication, liquids and artificial respiration
4. Other (describe): _________________________________________

**4.) After withdrawal of treatment, the patient is still breathing spontaneously. After several hours his oxygen saturation falls, he has a high temperature, but he still does not react, and his pupils are still dilated. What would you do in this situation?**

- 1. We would add oxygen and begin non-invasive ventilation, but we would not insert endotracheal intubation or connect him to a ventilator.
  2. We would add oxygen, reinsert the endotracheal intubation, and begin invasive mechanial ventilation.
  3. We would conduct palliative care measures (select)
     1. I would add oxygen
     2. we would take a sample for the laboratory and add antibiotics in order to protect other patients from possible infection
     3. we would give antipiretics, without additional oxygen or resuscitation measures
  4. Other (describe):_______________________________________________________
